# An Unusual Case of Swab Embolism

**DOI:** 10.1155/2014/405947

**Published:** 2014-09-29

**Authors:** N. G. Naidoo, D. Kahn, S. Mfolozi

**Affiliations:** ^1^Groote Schuur Hospital, Anzio Road, J45 Old Main Building, Cape Town 7925, South Africa; ^2^Vascular & Endovascular Surgery Unit, Department of Surgery, Medical School, University of Cape Town, Cape Town 7925, South Africa; ^3^Division of General Surgery, Department of Surgery, Medical School, University of Cape Town, Cape Town 7925, South Africa; ^4^Division of Forensic Medicine, Department of Pathology, University of Cape Town, Medical Campus, Falmouth Building, Cape Town 7925, South Africa

## Abstract

Intravascular foreign body embolism is an exceptionally uncommon problem. We report on an unusual case of a surgical swab embolism which occurred during a thoracic surgical procedure.

## 1. Case Report

A 47-year-old female presented with recurrent haemoptysis. She was on treatment for hypertension and type II diabetes mellitus. She had previously been treated for pulmonary tuberculosis on two separate occasions, including the mycobacterium avium complex. She was infected with the human immune-deficiency virus (HIV) and was on antiretroviral therapy with a CD4 count of 201 cells/mL.

The computed tomography (CT) scan of the chest revealed a destroyed right upper lobe with bronchiectasis and cavitation. The ventilation/perfusion scan revealed a nonfunctional right upper lobe.

The patient was subjected to a right posterolateral thoracotomy and a right bilobectomy. The procedure was complicated by an iatrogenic injury to the right superior pulmonary vein. There was significant haemorrhage following attempts to repair the injury which also involved compression with a small gauze (Ratex) swab. The patient became haemodynamically unstable, acidotic, hypothermic, and coagulopathic and required inotropic support. The operation was stopped and she was transferred to the intensive care unit (ICU) for stabilization. Concluding swab and instrument count in theatre identified a missing small gauze swab.

**Figure 1:**
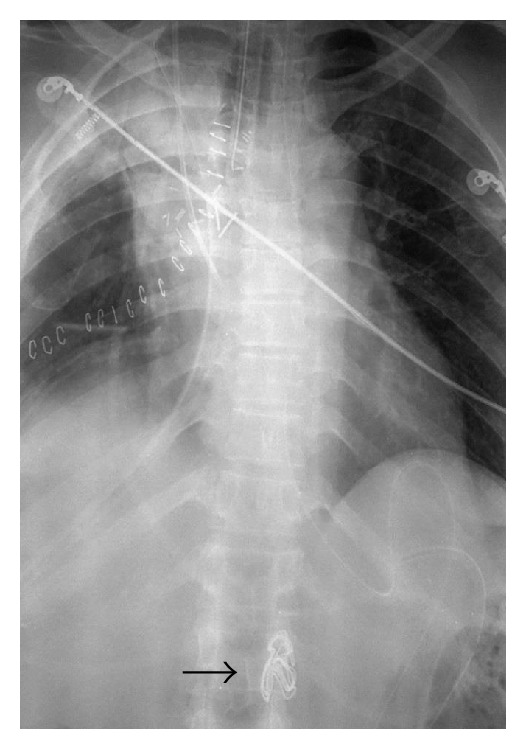
X-ray of chest showing location of the surgical swab (arrow).

**Figure 2:**
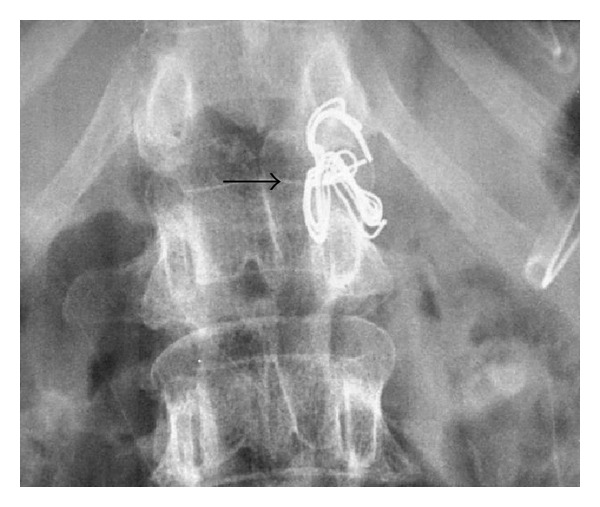
Magnified image showing swab between T12 and L1 vertebral levels (arrow).

In ICU, X-rays of the chest and abdomen showed a surgical swab in the aorta at the level of the diaphragm (Figures 1 and 2).

The vascular surgery unit was consulted and noted that the patient was responding poorly to resuscitation. She had absent leg pulses and established calf compartment rigidity bilaterally. Despite reservations regarding the merits of intervention, we were persuaded to attempt removal of the surgical swab using minimally invasive methods.

**Figure 3:**
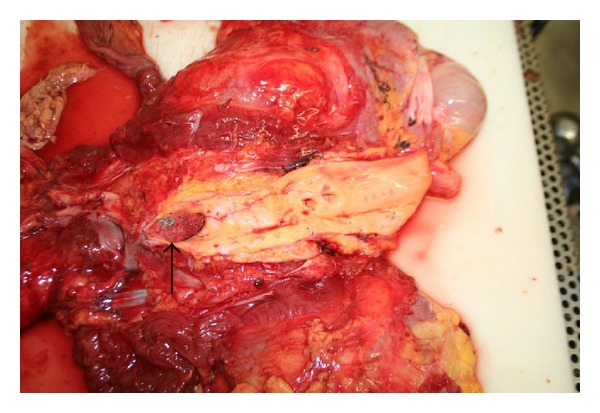
Post mortem pictures demonstrating swab impacted at the aortic bifurcation (black arrow).

In the operating theatre, the femoral vessels were exposed in the right groin and on-table angioimaging revealed a surgical swab impacted in the abdominal aorta above the level of the renal arteries. Removal of the swab using a filter retrieval catheter set was attempted. Unfortunately, we only managed to advance the swab down to the aortic bifurcation. The swab had consolidated into a solid, clotted mass making it virtually impossible to advance into the right iliac vessels (Figure 3).

The only option would have been an open conversion to surgically remove the swab. The patient deteriorated significantly and she progressed to multiorgan failure. Conversion to an open procedure to remove the swab was thought to be of too high risk. The decision, by consensus, was that any further attempts at salvage would be futile, and the procedure was terminated. The patient was returned to the ICU where she died a short while later.

## 2. Discussion

Intravascular foreign body embolism is exceptionally rare and may be classified as iatrogenic, occurring in the course of endovascular therapy [1], or trauma related (e.g., bullet embolism) [2]. The exponential increase in endovascular procedures has been accompanied by an increase in iatrogenic endovascular device or device fragment embolism. These have included stents, catheter tips, guide-wire fragments, inferior vena cava filters or filter struts, embolization coils, intravascular ultrasound probes, pacemaker leads, and septal occluders. The frequency of bullet embolism complicating a gunshot wound is rare (<0.4%).

Venous embolism has been described more commonly than arterial embolism (estimated ratio 4 : 1). The majority of these cases were asymptomatic. Most of these foreign bodies were retrieved with snares and less commonly with retrieval forceps, balloon catheters, or ureteral stone baskets [1, 3, 4]. Larger objects (septal occluders and bullets) generally required an open approach. Alternatively, large objects may be trawled by endovascular means into a vessel more accessible for open retrieval, for example, the femoral vessels. Case reports of open retrieval are often accompanied by a significant mortality approaching 38%.

We describe an unusual case of surgical swab embolism following thoracic surgery (lobectomy). The natural taper of the abdominal aorta resulted in impaction at the level of the renal arteries resulting in significant mesenteric, renal, and lower extremity ischaemia (Rutherford grade III acute limb ischaemia). We attempted, using endovascular means, to advance the swab into a more accessible vessel for retrieval.

## 3. Conclusion

The use of small gauze swabs in open cavity surgery, especially laparotomy, has been repeatedly challenged. These swabs tend to consolidate into a small clotted “peach seed” that tends to lose its way into the abdomen as a retained swab with significant postoperative sequelae: intra-abdominal sepsis, fistulae, and so forth. Only large mopping swabs with an attached colored tape tag should be used in open cavity surgery to avoid not only the conventionally recognized complications related to retained swabs but also bizarre complications including the case described in this report.
